# Angiotensin‐converting enzyme inhibitors and angiotensin II receptor blockers are not associated with severe COVID‐19 infection in a multi‐site UK acute hospital trust

**DOI:** 10.1002/ejhf.1924

**Published:** 2020-07-07

**Authors:** Daniel M. Bean, Zeljko Kraljevic, Thomas Searle, Rebecca Bendayan, O'Gallagher Kevin, Andrew Pickles, Amos Folarin, Lukasz Roguski, Kawsar Noor, Anthony Shek, Rosita Zakeri, Ajay M. Shah, James T.H. Teo, Richard J.B. Dobson

**Affiliations:** ^1^ Department of Biostatistics and Health Informatics Institute of Psychiatry, Psychology and Neuroscience, King's College London London UK; ^2^ Health Data Research UK London University College London London UK; ^3^ NIHR Biomedical Research Centre at South London and Maudsley NHS Foundation Trust and King's College London London UK; ^4^ King's College Hospital NHS Foundation Trust London UK; ^5^ School of Cardiovascular Medicine & Sciences King's College London British Heart Foundation Centre of Excellence London UK; ^6^ Institute of Health Informatics University College London London UK; ^7^ NIHR Biomedical Research Centre University College London Hospitals NHS Foundation Trust London UK; ^8^ Department of Clinical Neuroscience Institute of Psychiatry, Psychology and Neuroscience, King's College London London UK

**Keywords:** COVID‐19, Angiotensin‐converting enzyme inhibitors, Hypertension, Disease outcome

## Abstract

**Aims:**

The SARS‐CoV‐2 virus binds to the angiotensin‐converting enzyme 2 (ACE2) receptor for cell entry. It has been suggested that angiotensin‐converting enzyme inhibitors (ACEi) and angiotensin II receptor blockers (ARB), which are commonly used in patients with hypertension or diabetes and may raise tissue ACE2 levels, could increase the risk of severe COVID‐19 infection.

**Methods and results:**

We evaluated this hypothesis in a consecutive cohort of 1200 acute inpatients with COVID‐19 at two hospitals with a multi‐ethnic catchment population in London (UK). The mean age was 68 ± 17 years (57% male) and 74% of patients had at least one comorbidity. Overall, 415 patients (34.6%) reached the primary endpoint of death or transfer to a critical care unit for organ support within 21 days of symptom onset. A total of 399 patients (33.3%) were taking ACEi or ARB. Patients on ACEi/ARB were significantly older and had more comorbidities. The odds ratio for the primary endpoint in patients on ACEi and ARB, after adjustment for age, sex and co‐morbidities, was 0.63 (95% confidence interval 0.47–0.84, *P* < 0.01).

**Conclusions:**

There was no evidence for increased severity of COVID‐19 in hospitalised patients on chronic treatment with ACEi or ARB. A trend towards a beneficial effect of ACEi/ARB requires further evaluation in larger meta‐analyses and randomised clinical trials.

## Introduction

The COVID‐19 pandemic is a major medical and socioeconomic challenge with at least 3 million confirmed cases to date. Data on the clinical characteristics of patients who require hospital admission for COVID‐19 from China, Italy and the US consistently show that patients with cardiovascular comorbidities are over‐represented and may have an increased risk of severe COVID‐19 disease.^1^, ^2^, ^3^ The reasons underlying the increased incidence of severe COVID‐19 infection in those with comorbidities such as hypertension, diabetes and other cardiovascular conditions are unknown.

The SARS‐CoV‐2 virus requires the binding of its viral surface spike protein to the angiotensin‐converting enzyme 2 (ACE2) receptor expressed on epithelial cells in order to be internalised and then undergo replication.^4^ Previous studies suggest that the expression of ACE2 may be increased by chronic treatment with angiotensin‐converting enzyme inhibitors (ACEi) or angiotensin II receptor blockers (ARB).^5^ As such, it has been hypothesized that treatment with ACEi or ARB could increase the likelihood of SARS‐CoV‐2 binding and entry into epithelial or other cells.^6^ Furthermore, it is hypothesised that such a mechanism could account for the increased incidence of severe COVID‐19 infection among patients with cardiovascular comorbidities, who are frequently treated with ACEi/ARB.^6^ Whether or not treatment with ACEi/ARB increases the risk of severe COVID‐19 disease is a very important question in view of the large numbers of patients potentially on these drugs, especially in western countries with older populations. The issue is controversial because ACEi/ARB may potentially be beneficial in severe lung injury by reducing activation of the renin–angiotensin system (RAS).^7^, ^8^, ^9^, ^10^ Furthermore, increased levels of ACE2 itself have been shown to be protective during severe lung injury.^11^, ^12^ The potential effect of ACEi and ARB during infection with SARS‐CoV‐2 therefore requires urgent clarification.

We tested for association between treatment with ACEi/ARB and disease severity in a consecutive series of 1200 patients with COVID‐19 disease admitted to two UK hospitals, King's College Hospital and Princess Royal University Hospital, that have been at the epicentre of the pandemic in London. We used an established and validated informatics pipeline to allow rapid evaluation of this important question during the pandemic.

## Methods

This project operated under London South East Research Ethics Committee approval (reference 18/LO/2048) granted to the King's Electronic Records Research Interface (KERRI); specific work on COVID‐19 research was reviewed with expert patient input on a virtual committee with Caldicott Guardian oversight.

### Study design

The study cohort was defined as all adult inpatients testing positive for COVID‐19 by reverse transcriptase polymerase chain reaction at King's College Hospital and Princess Royal University Hospital from 1 March to 13 April 2020. Only symptomatic patients who required inpatient admission were included. Presenting symptoms included but were not limited to fever, cough, dyspnoea, myalgia, chest pain, or delirium. The primary endpoint was defined as death or admission to a critical care unit for organ‐support within 21 days of symptom onset. Data were collected for a range of clinical and demographic parameters (*Table* *1*). To ascertain chronic treatment with ACEi, ARB and other relevant medications, we captured information from clinical notes, outpatient clinic letters and inpatient medication orders. If a drug was a regular medication in the community but withheld on admission, we considered this to be on chronic treatment. The primary endpoint was manually verified by clinician review of the electronic health record.

**Table 1:** Characteristics of the 1200 patients positive for COVID‐19 at Princess Royal University Hospital and King's College Hospital, London, UK

	All Patients	On ACEi/ARB	Not on ACEi/ARB	*P*‐value
*n*	1200	399	801	
Age, years, mean (SD)	67.96 (17.07)	73.02 (13.46)	65.45 (18.1)	<0.001
Sex				
Male	686 (57.2%)	231 (57.9%)	455 (56.8%)	1.0
Female	514 (42.8%)	168 (42.1%)	346 (43.2%)	1.0
Ethnicity				
Caucasian	512 (42.7%)	170 (42.6%)	342 (42.7%)	1.0
Black	310 (25.8%)	105 (26.3%)	205 (25.6%)	1.0
Asian	58 (4.8%)	21 (5.3%)	37 (4.6%)	1.0
Unknown/mixed/other	320 (26.7%)	103 (25.8%)	217 (27.1%)	1.0
Comorbidity				
HTN	645 (53.8%)	339 (85.0%)	306 (38.2%)	<0.001
Diabetes	418 (34.8%)	215 (53.9%)	203 (25.3%)	<0.001
HF	107 (8.9%)	65 (16.3%)	42 (5.2%)	<0.001
IHD	160 (13.3%)	83 (20.8%)	77 (9.6%)	<0.001
COPD	121 (10.1%)	42 (10.5%)	79 (9.9%)	1.0
Asthma	169 (14.1%)	58 (14.5%)	111 (13.9%)	1.0
CKD	206 (17.2%)	108 (27.1%)	98 (12.2%)	<0.001
Previous stroke/TIA	235 (19.6%)	112 (28.1%)	123 (15.4%)	<0.001
BMI, kg/m^2^, mean (SD)	26.3 (8.7)	27.0 (8.5)	25.8 (8.4)	1.0
BMI ≥30 kg/m^2^	182 (15.2%)	69 (17.3%)	113 (14.1%)	1.0
No. of comorbidities				
0	310 (25.8%)	19 (4.8%)	291 (36.3%)	<0.001
1	283 (23.6%)	73 (18.3%)	210 (26.2%)	0.08
>1	607 (50.6%)	307 (76.9%)	300 (37.5%)	<0.001
Drugs				
ACEi	260 (21.7%)	260 (65.2%)	0 (0.0%)	
ARB	147 (12.2%)	147 (36.8%)	0 (0.0%)	
Statin	472 (39.3%)	268 (67.2%)	204 (25.5%)	<0.001
Beta‐blocker	337 (28.1%)	184 (46.1%)	153 (19.1%)	<0.001
Vital signs				
Systolic BP, mmHg, mean (SD)	124 (27)	126 (28)	123 (26)	0.17
Diastolic BP, mmHg, mean (SD)	71 (18)	71 (18)	71 (18)	1.0
Primary endpoint by 21 days				
Death or critical care admission	415 (34.6%)	127 (31.8%)	288 (36.0%)	1.0
Death	288 (24.0%)	106 (26.6%)	182 (22.7%)	1.0
Critical care admission and alive	127 (10.6%)	21 (5.3%)	106 (13.2%)	<0.01

Data were available on all patients except for ethnicity (n = 925), systolic BP (n = 1120), diastolic BP (n = 1120), BMI (n = 621).

ACEi, angiotensin‐converting enzyme inhibitor; ARB, angiotensin II receptor blocker; BMI, body mass index; BP, blood pressure; CKD, chronic kidney disease; COPD, chronic obstructive pulmonary disease; HF, heart failure; HTN, hypertension; IHD, ischaemic heart disease; SD, standard deviation; TIA, transient ischaemic attack.

*P*‐value comparing the group on ACEi /ARB vs. not on ACEi/ARB with Bonferroni correction for multiple testing. Continuous variables compared with *t*‐test, binary variables compared with Chi‐squared test.

### Data processing

The data (demographic, emergency department letters, discharge summaries, clinical notes, radiology reports, medication orders, lab results) was retrieved and analysed in near real‐time from the structured and unstructured components of the electronic health record using a variety of well‐validated natural language processing (NLP) informatics tools belonging to the CogStack ecosystem,^13^ namely DrugPipeline,^14^ MedCAT^15^ and MedCATTrainer.^16^ The CogStack NLP pipeline captures negation, synonyms, and acronyms for medical SNOMED‐CT concepts as well as surrounding linguistic context using deep learning and long short‐term memory networks. DrugPipeline was used to annotate medications and MedCAT produced unsupervised annotations for all SNOMED‐CT concepts under parent terms Clinical Finding, Disorder, Organism, and Event with disambiguation, pre‐trained on MIMIC‐III.^17^ Further supervised training improved detection of annotations and meta‐annotations such as experiencer (is the concept annotated experienced by the patient or other), negation (is the concept annotated negated or not) and temporality (is the concept annotated in the past or present) with MedCATTrainer. Meta‐annotations for hypothetical and experiencer were merged into irrelevant meaning that any concept annotated as either hypothetical or where the experiencer was not the patient was annotated as irrelevant. Performance of the MedCAT NLP pipeline for disorders mentioned in the text was evaluated on 5617 annotations for 265 documents by a domain expert (JTHT) and F1, precision and recall recorded. Additional full case review for correct subsequent diagnosis assignment was performed by three clinicians (JTHT, KOG, RZ) for key comorbidities. The performance of DrugPipeline has previously been described.^14^ Manual review of 100 detections gave F1 = 0.91 for exclusion of drug allergies by DrugPipeline.

### Statistical analysis

In order to investigate the association between ACEi/ARB and disease severity measured as critical care admission or death, we performed a series of logistic regressions. In a first step, we explored independently the association for ACEi/ARB (baseline model). In a second step, we adjusted the model for age and sex (Model 1). Then, we additionally adjusted for hypertension (Model 2) and finally, additionally adjusted for other comorbidities, i.e. diabetes, ischaemic heart disease, heart failure and chronic kidney disease (Model 3). We also explored the independent association for hypertension following the same modelling approach. In addition, we assessed the robustness to unmeasured confounders of the fully adjusted estimate of ACEi/ARB effect using the e‐value approach, which are defined as the minimum strength of association on the risk ratio scale that an unmeasured confounder would need to have with both the treatment assignment and the outcome to fully explain away a specific treatment–outcome association, conditional on the measured covariates.^18^ Sensitivity analyses were performed (i) requiring at least two detections of medication for positive exposure; (ii) using only structured data on in‐hospital medication orders; (iii) ignoring our 21‐day window for medications; and (iv) testing sensitivity to unmeasured confounders.

### Role of the funding source

The funders had no role in study design, data collection and analysis, decision to publish, or preparation of the manuscript.

## Results

**Figure 1:**
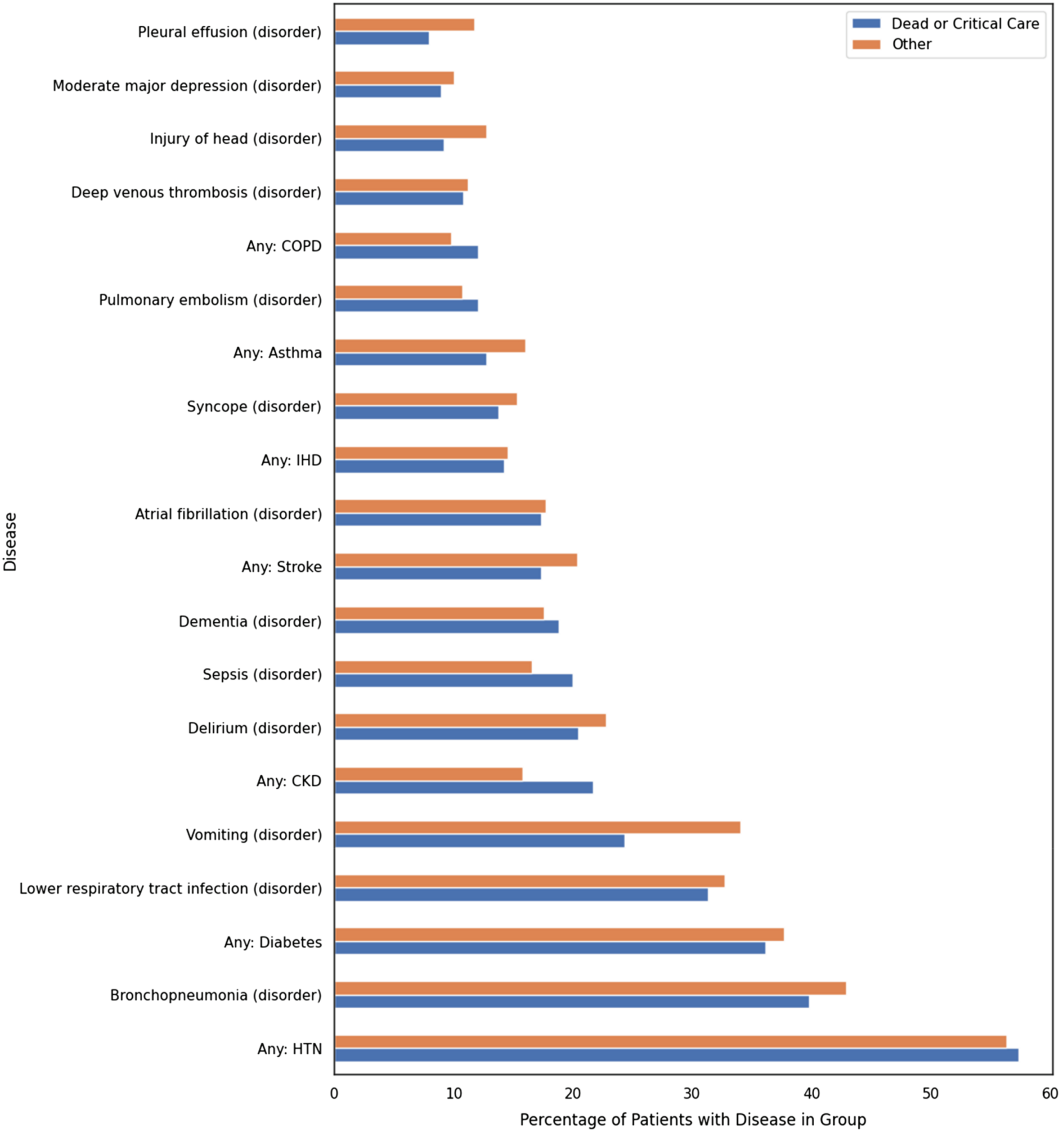
Distribution of disorders between patients achieving the primary outcome (death or critical care admission) and those not achieving it by 21 days after symptom onset. The percentage of patients that have a positive mention of a disorder in each of the two groups is shown. All diseases were extracted from free‐text using CogStack and MedCAT. Only medical concept annotations with F1 >80%, more than 10 annotated samples and present in at least 10% of either group are shown. Disease names that start “Any:” are aggregate concepts for multiple specific conditions that are used in our analysis. CKD, chronic kidney disease; COPD, chronic obstructive pulmonary disease; HTN, hypertension; IHD, ischaemic heart disease.

Our total cohort consisted of 1200 confirmed positive symptomatic inpatients aged 63 ± 20 years with 52% being male (*Table* *1*). The patients were of diverse ethnicities with over 30% from minority ethnic groups. Nearly 75% of patients had one or more comorbidities. The commonest comorbidities were hypertension (51.2%), diabetes (30.2%), chronic kidney disease (17.2%), and ischaemic heart disease or heart failure (22.2%). Overall, 15.2% of patients had a body mass index >30 kg/m^2^. A total of 399 patients (33.2%) were on chronic treatment with ACEi or ARB; 415 of the 1200 patients (34.6%) required admission to the critical care unit or had died within 21 days of symptom onset. Among patients who achieved the primary endpoint (death or critical care admission), the percentage who had positive mentions for various disorders derived via the NLP for medical concept annotations with F1 >80% and more than 10 annotated mentions, as compared to those not achieving the primary endpoint, is shown in *Figure* *1*. The performance of the NLP pipeline is shown in online supplementary *Figure S1*. Manual validation of the presence of comorbidities was performed in a sample of 200 patients and showed excellent performance, for example a false positive rate of 1% for hypertension and 0% for diabetes.

**Figure 2:**
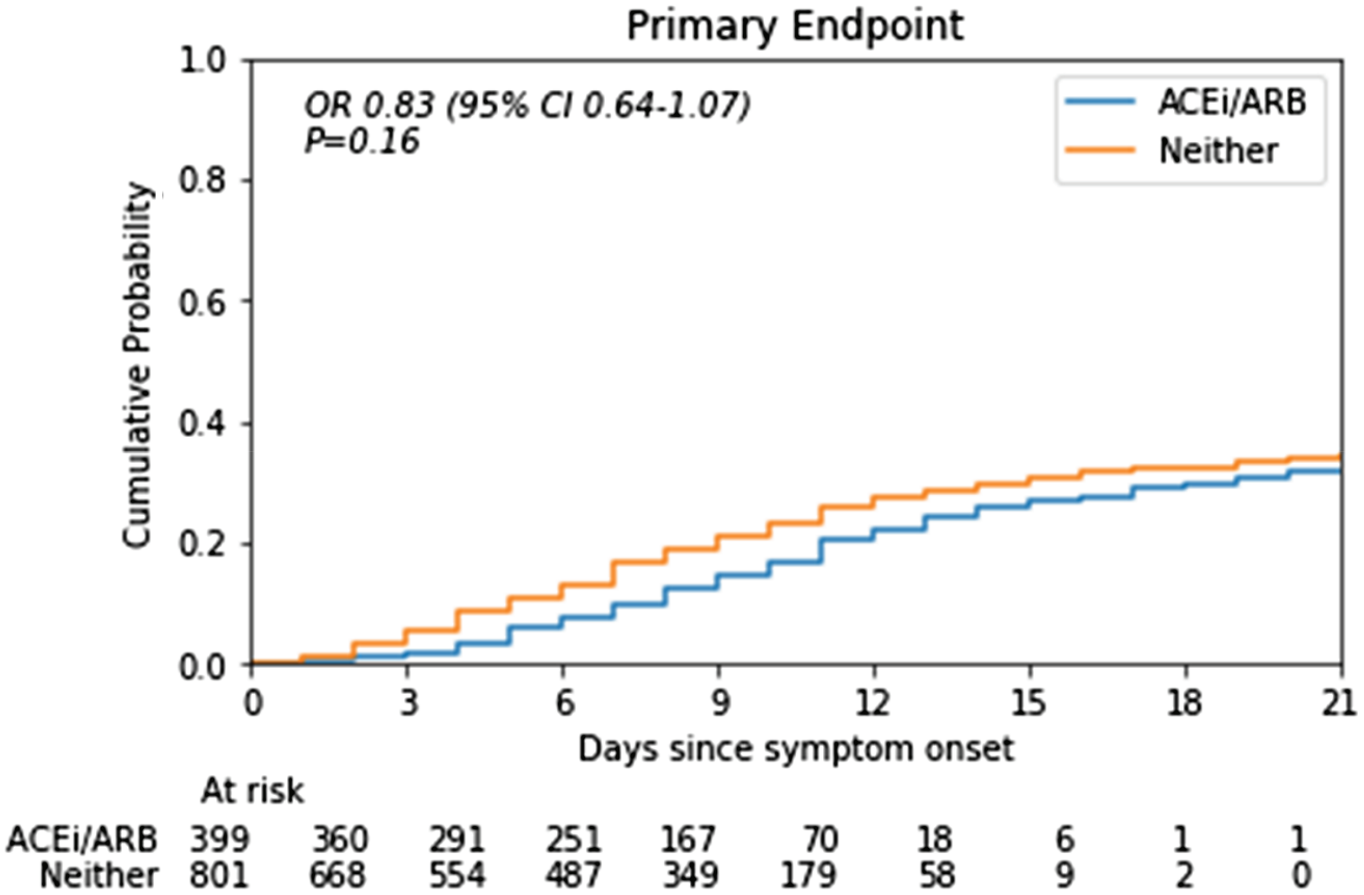
Kaplan–Meier curves for the primary endpoint in COVID‐19 patients on chronic treatment with angiotensin‐converting enzyme inhibitors (ACEi) or angiotensin II receptor blockers (ARB) vs. those not on these drugs. The unadjusted odds ratio (OR) for the primary endpoint for those on ACEi/ARB was 0.83 (*P* = 0.16); the adjusted OR was 0.63 (*P* < 0.01). CI, confidence interval.

We next compared the outcome of patients on chronic treatment with ACEi/ARB vs. those not on these agents. The group on ACEi/ARB were significantly older but had a similar male/female split and a similar ethnicity profile to those not on ACEi/ARB (*Table* *1*). The body mass index was similar between groups. There was a greater proportion of patients with cardiovascular comorbidities (hypertension, diabetes, heart failure, ischaemic heart disease) and chronic kidney disease in the group on ACEi/ARB than those not taking these drugs, as would be expected. Therefore, the patients on ACEi/ARB had a higher prevalence of factors associated with worse outcome of COVID‐19 disease in prior studies.^1^, ^2^, ^3^ The ACEi/ARB group also had higher rates of treatment with beta‐blockers and statins than those not on ACEi/ARB, consistent with their higher rates of cardiovascular morbidities. *Figure* *2* shows Kaplan–Meier curves for the primary endpoint in patients on ACEi/ARB and those not on these drugs.

To assess the independent effect of ACEi/ARB on the primary outcome, we first performed an unadjusted logistic regression analysis. This indicated that the likelihood of a severe outcome was similar in individuals on ACEi/ARB as compared to those not on these drugs, with an odds ratio (OR) of 0.83 [95% confidence interval (CI) 0.64–1.07] (baseline model in *Table* *2*). However, after adjustments for age and sex (Model 1 in *Table* *2*), the likelihood of severe disease was significantly lower in those on ACEi/ARBs (OR 0.70, 95% CI 0.53–0.91; *P* < 0.01). Additional adjustment for hypertension (Model 2 in *Table* *2*) and for the other major comorbidities, including diabetes, chronic kidney disease, and ischaemic heart disease/heart failure (Model 3 in *Table* *2*), had a modest further effect. The OR for the primary outcome in Model 3 was 0.63 (95 CI 0.47–0.84; *P* < 0.01). Online supplementary *Table S1* shows the OR and *P*‐values for all variables in each model. Male patients were found to have a higher likelihood of severe disease in Model 3 (OR 1.50, 95% CI 1.17–1.93; *P* <0.01).

**Table 2:** Summary of odds ratios for angiotensin‐converting enzyme inhibitor and/or angiotensin II receptor blocker drug use and the primary endpoint

Model	Adjustments	OR (95% CI) ACEi/ARB vs. neither drug	*P*‐value
Baseline	–	0.83 (0.64–1.07)	0.16
Model 1	Age, sex	0.70 (0.53–0.91)	<0.01
Model 2	Age, sex, hypertension	0.64 (0.48–0.86)	<0.01
Model 3	Age, sex, hypertension, diabetes mellitus, chronic kidney disease, ischaemic heart disease, heart failure	0.63 (0.47–0.84)	<0.01

ACEi, angiotensin‐converting enzyme inhibitor; ARB, angiotensin II receptor blocker; CI, confidence interval; OR, odds ratio.

OR and *P*‐values calculated from logistic regressions.

We also examined the independent association between hypertension and disease severity. The results showed that individuals with hypertension had a similar likelihood of suffering a severe outcome as those without hypertension, either in unadjusted models (OR 1.25, 95% CI 0.98–1.59; *P* = 0.069) or in models adjusted for age and gender (OR 1.03, 95% CI 0.80–1.32; *P* = 0.83).

Sensitivity analyses were performed using criteria for ACEi exposure that were either more strict (requiring multiple mentions in the clinical notes or using only in‐hospital medication orders as evidence) or less strict (including any mention of ACEi treatment even outside a 21‐day window from onset of symptoms). In all cases, the estimates of the impact of ACEi treatment were consistent with those in *Table* *2*. In analysis requiring at least two mentions of chronic treatment with ACEi/ARB, we found that this was significant in the uncorrected baseline model (a lower OR). We estimated an e‐value of 1.82, which suggests that the estimate, though clearly significant, could yet be vulnerable to possible confounders not yet included.

## Discussion

This study in a large consecutive cohort of 1200 patients in the UK suggests that chronic treatment with ACEi and ARB is not associated with an increase in severe outcome of COVID‐19 disease, defined as death or admission to a critical care unit. The hypothetical relationship between treatment with ACEi/ARB and severe COVID‐19 disease has been intensely debated.^6^, ^7^, ^8^, ^9^, ^10^ There are theoretical mechanisms whereby chronic treatment with ACEi/ARB might increase propensity to SARS‐CoV‐2 infection as well as other mechanisms whereby treatment with these agents might be beneficial. It is a particularly important question because chronic treatment with ACEi/ARB is of proven benefit in conditions such as hypertension, diabetes, chronic kidney disease and heart failure, and an unwarranted cessation of therapy in patients with these conditions as a result of the COVID‐19 pandemic could have serious long‐term detrimental effects.

The general clinical characteristics and the rates of severe outcome of the patients in our study were broadly similar to those that have been described in recent large series from Italy and the USA.^1^, ^2^, ^3^ We found that patients who were on chronic treatment with ACEi/ARB had many demographic and comorbidity features that have been associated in previous studies with worse outcome in COVID‐19 disease, such as an older age and a higher prevalence of hypertension, diabetes, heart failure and other morbidities.^1^, ^2^, ^3^ Treatment with ACEi/ARB was nevertheless not associated with an increase in rates of severe outcomes, with or without adjustment for age, sex and comorbidities. A number of very recent studies have now also reported on the relationship between ACEi/ARB and outcome of COVID‐19 disease in hospitalised patients. In a single centre study from Wuhan in which only 115 of 1178 patients (<10%) were taking ACEi/ARB, the authors did not find any relationship between these drugs and outcome^19^; the data are however limited by the low numbers on ACEi/ARB and potential confounding by other factors. A second report from China was a retrospective multi‐centre study including 1128 patients but again had only 188 patients (16.6%) on treatment with ACEi/ARB.^20^ This study suggested that treatment with ACEi/ARB was associated with a lower rate of severe outcome with COVID‐19 infection. Mancia *et al*.^21^ reported a case control series from Italy in which 617 patients had severe COVID‐19 disease among 6272 SARS‐CoV‐2 positive cases, and the rates of ACEi/ARB usage were higher. These authors found no association between ACEi/ARB and the likelihood of infection or fatal disease. However, no data on ethnicity were included in this study.^21^ At the time of submission, our study was the first to be conducted on an ethnically mixed population in the western world and to include significant proportions of both White and minority ethnic (Black, Asian) patients. The rates of usage of ACEi/ARB in our study (33.2%) are in line with those expected in well‐treated patients with comorbidities and are therefore, in principle, more applicable to patients in Europe and the Americas. Ethnicity is a very pertinent issue in this regard due to the recognised ethnicity‐related differences in response to drugs affecting the RAS.^21^, ^22^ Of relevance, the ethnicity profiles of the patients on ACEi/ARB in our study were similar to those not taking these drugs. A recent study from New York also reports on a multi‐ethnic population among whom 1002 patients developed severe COVID‐19 illness.^23^ These authors found no evidence of an increased risk of severe COVID‐19 in patients taking ACEi or ARB. Finally, an analysis of patients with heart failure reports no association between ACEi/ARB treatment and the concentrations of plasma ACE2.^24^ Although the relevance of plasma ACE2 to susceptibility to SARS‐CoV‐2 (which binds to cell surface ACE2)^4^ is unclear, this study also fails to provide evidence in support of the theoretical risks of ACEi/ARB with respect to COVID‐19.

In the current study, when we adjusted for age, sex and comorbidities in logistic regression analyses, the OR for a severe outcome was significantly lower in patients on ACEi/ARB than those not on these agents. This suggestion of a favourable association of treatment with ACEi/ARB and less severe outcome in COVID‐19 disease would be consistent with the hypothesised beneficial effects of inhibition of RAS activation in patients with severe lung injury or acute respiratory distress syndrome.^7^, ^8^ However, due to the possibility of unmeasured confounding factors, the confirmation of a potential therapeutic benefit of treatment with ACEi/ARB in COVID‐19 disease would require further studies and randomised controlled trials.

This study used an NLP approach to perform very rapid analysis of high volume, unstructured real‐world clinical data. This however introduces the possibility of missing circumlocutory mentions of disease, symptoms, or medications. We mitigated against this by manually validating annotations in a subset of records and also verifying drug treatments against inpatient electronic prescription data. Moreover, we performed sensitivity analyses to test the impact of different criteria to define the ACEi/ARB exposed cohort on our results, and found that the OR remained <1.0 and significant for ACEi/ARB exposure in all adjusted analyses. We therefore consider our analysis pipeline to be robust to specific details of the pipeline that are not clinically relevant. However, we did find that the estimated OR may be sensitive to unmeasured confounding, which suggests caution in the interpretation of any protective effect, and the need for replication in a larger sample remains.

Our study has some potential limitations. Although the patients and data were prospectively collected, the analyses were retrospective. The study was conducted on two hospital sites in a single geographical, albeit ethnically mixed, locus in the UK over a relatively short follow‐up period. However, the duration of follow‐up is sufficient to accurately detect early severe outcomes based on the data from multiple studies during the current pandemic. We used the covariates identified as important in the previous large case series on COVID‐19,^1^, ^2^, ^3^ including age, sex and common comorbidities, to adjust our analyses. However, it is possible that other unmeasured confounders could have influenced the results. For example, the patients on chronic ACEi/ARB treatment were also more frequently treated with statins than those not on these drugs, which could suggest that their medical conditions were generally better managed. However, the ACEi/ARB group was also older and had higher rates of hypertension, diabetes and multiple morbidities, making it unlikely that these patients were physiologically healthier. Our study was performed in patients with COVID‐19 who required hospitalisation; the effect of chronic treatment with ACEi/ARB on less severe infection with SARS‐CoV‐2 in the non‐hospital setting requires further study. Whether the current results are applicable to other global populations, such as in Africa, will also require additional study.

In summary, the results of this study in 1200 patients show no evidence of a detrimental effect of chronic treatment with ACEi/ARB in patients presenting with severe COVID‐19 infection. Patients on treatment with ACEi/ARB should continue these drugs during their COVID‐19 illness as per current European Society of Cardiology guidelines.^25^

## Supporting information


**Table S1.** Odds ratios and *P*‐values for all variables and primary endpoint.
**Figure S1**. Performance of the CogStack and MedCAT NLP pipeline in detecting disease mentions within the electronic health record text.

## References

[1] Guan WJ , Ni ZY , Hu Y , Liang WH , Ou CQ , He JX , Liu L , Shan H , Lei CL , Hui DSC , Du B , Li LJ , Zeng G , Yuen KY , Chen RC , Tang CL , Wang T , Chen PY , Xiang J , Li SY , Wang JL , Liang ZJ , Peng YX , Wei L , Liu Y , Hu YH , Peng P , Wang JM , Liu JY , Chen Z , Li G , Zheng ZJ , Qiu SQ , Luo J , Ye CJ , Zhu SY , Zhong NS ; China Medical Treatment Expert Group for Covid‐19 . Clinical characteristics of coronavirus disease 2019 in China. N Engl J Med 2020;382:1708–1720. 32109013 10.1056/NEJMoa2002032 PMC7092819

[2] Grasselli G , Zangrillo A , Zanella A , Antonelli M , Cabrini L , Castelli A , Cereda D , Coluccello A , Foti G , Fumagalli R , Iotti G , Latronico N , Lorini L , Merler S , Natalini G , Piatti A , Ranieri MV , Scandroglio AM , Storti E , Cecconi M , Pesenti A ; COVID‐19 Lombardy ICU Network . Baseline characteristics and outcomes of 1591 patients infected with SARS‐CoV‐2 admitted to ICUs of the Lombardy Region, Italy. JAMA 2020;323:1574–1581. 32250385 10.1001/jama.2020.5394 PMC7136855

[3] Richardson S , Hirsch JS , Narasimhan M , Crawford JM , McGinn T , Davidson KW ; Northwell COVID‐19 Research Consortium . Presenting characteristics, comorbidities, and outcomes among 5700 patients hospitalized with COVID‐19 in the New York City area. JAMA 2020;323:2052–2059. 32320003 10.1001/jama.2020.6775 PMC7177629

[4] Hoffmann M , Kleine‐Weber H , Schroeder S , Krüger N , Herrler T , Erichsen S , Schiergens TS , Herrler G , Wu N‐H , Nitsche A , Müller MA , Drosten C , Pöhlmann S . SARS‐CoV‐2 cell entry depends on ACE2 and TMPRSS2 and is blocked by a clinically proven protease inhibitor. Cell 2020;181:271–280.e8. 32142651 10.1016/j.cell.2020.02.052 PMC7102627

[5] Vuille‐dit‐Bille RN , Camargo SM , Emmenegger L , Sasse T , Kummer E , Jando J , Hamie QM , Meier CF , Hunziker S , Forras‐Kaufmann Z , Kuyumcu S , Fox M , Schwizer W , Fried M , Lindenmeyer M , Götze O , Verrey F . Human intestine luminal ACE2 and amino acid transporter expression increased by ACE‐inhibitors. Amino Acids 2015;47:693–705. 25534429 10.1007/s00726-014-1889-6

[6] Zheng YY , Ma YT , Zhang JY , Xie X . COVID‐19 and the cardiovascular system. Nat Rev Cardiol 2020;17:259–260. 32139904 10.1038/s41569-020-0360-5 PMC7095524

[7] Vaduganathan M , Vardeny O , Michel T , McMurray JJ , Pfeffer MA , Solomon SD . Renin‐angiotensin‐aldosterone system inhibitors in patients with Covid‐19. N Engl J Med 2020;382:1653–1659. 32227760 10.1056/NEJMsr2005760 PMC7121452

[8] Kuster GM , Pfister O , Burkard T , Zhou Q , Twerenbold R , Haaf P , Widmer AF , Osswald S . SARS‐CoV2: should inhibitors of the renin‐angiotensin system be withdrawn in patients with COVID‐19? Eur Heart J 2020;41:1801–1803. 32196087 10.1093/eurheartj/ehaa235 PMC7184407

[9] Tomasoni D , Italia L , Adamo M , Inciardi RM , Lombardi CM , Solomon SD , Metra M . COVID‐19 and heart failure: from infection to inflammation and angiotensin II stimulation. Searching for evidence from a new disease. Eur J Heart Fail 2020;22:957–966. 32412156 10.1002/ejhf.1871 PMC7273093

[10] Danser AH , Epstein M , Batlle D . Renin‐angiotensin system blockers and the COVID‐19 pandemic: at present there is no evidence to abandon renin‐angiotensin system blockers. Hypertension 2020;75:1382–1385. 32208987 10.1161/HYPERTENSIONAHA.120.15082 PMC7225046

[11] Imai Y , Kuba K , Rao S , Huan Y , Guo F , Guan B , Yang P , Sarao R , Wada T , Leong‐Poi H , Crackower MA , Fukamizu A , Hui CC , Hein L , Uhlig S , Slutsky AS , Jiang C , Penninger JM . Angiotensin‐converting enzyme 2 protects from severe acute lung failure. Nature 2005;436:112–116. 16001071 10.1038/nature03712 PMC7094998

[12] Kuba K , Imai Y , Rao S , Gao H , Guo F , Guan B , Huan Y , Yang P , Zhang Y , Deng W , Bao L , Zhang B , Liu G , Wang Z , Chappell M , Liu Y , Zheng D , Leibbrandt A , Wada T , Slutsky AS , Liu D , Qin C , Jiang C , Penninger JM . A crucial role of angiotensin converting enzyme 2 (ACE2) in SARS coronavirus–induced lung injury. Nat Med 2005;11:875–879. 16007097 10.1038/nm1267 PMC7095783

[13] Jackson R , Kartoglu I , Stringer C , Gorrell G , Roberts A , Song X , Wu H , Agrawal A , Lui K , Groza T , Lewsley D , Northwood D , Folarin A , Stewart R , Dobson R . CogStack ‐ experiences of deploying integrated information retrieval and extraction services in a large National Health Service Foundation Trust hospital. BMC Med Inform Decis Mak 2018;18:47. 29941004 10.1186/s12911-018-0623-9 PMC6020175

[14] Bean DM , Teo J , Wu H , Oliveira R , Patel R , Bendayan R , Shah AM , Dobson RJB , Scott PA . Semantic computational analysis of anticoagulation use in atrial fibrillation from real world data. PLoS One 2019;14 e0225625. 31765395 10.1371/journal.pone.0225625 PMC6876873

[15] Kraljevic Z , Bean D , Mascio A , Roguski L , Folarin A , Roberts A , Bendayan R , Dobson R. MedCAT – medical concept annotation tool. arXiv, 2019. https://arxiv.org/abs/1912.10166 (8 June 2020).

[16] Searle T , Kraljevic Z , Bendayan R , Bean D , Dobson R . MedCATTrainer: a biomedical free text annotation interface with active learning and research use case specific customisation. Proceedings of the 2019 Conference on Empirical Methods in Natural Language Processing and the 9th International Joint Conference on Natural Language Processing (EMNLP‐IJCNLP): System Demonstrations. Stroudsburg, PA: Association for Computational Linguistics; 2019. p. 139–144.

[17] Johnson AE , Pollard TJ , Shen L , Lehman L‐WH , Feng M , Ghassemi M , Moody B , Szolovits P , Celi LA , Mark RG . MIMIC‐III, a freely accessible critical care database. Sci Data 2016;3:160035. 27219127 10.1038/sdata.2016.35 PMC4878278

[18] Linden A , Mathur MB , VanderWeele TJ . Conducting sensitivity analysis for unmeasured confounding in observational studies using E‐values: the evalue package. Stata J 2020;20:162–175.

[19] Li J , Wang X , Chen J , Zhang H , Deng A . Association of renin‐angiotensin system inhibitors with severity or risk of death in patients with hypertension hospitalized for coronavirus disease 2019 (COVID‐19) infection in Wuhan, China. JAMA Cardiol 2020 Apr 23. 10.1001/jamacardio.2020.1624 [Epub ahead of print]. PMC7180726 32324209

[20] Zhang P , Zhu L , Cai J , Lei F , Qin JJ , Xie J , Liu YM , Zhao YC , Huang X , Lin L , Xia M , Chen MM , Cheng X , Zhang X , Guo D , Peng Y , Ji YX , Chen J , She ZG , Wang Y , Xu Q , Tan R , Wang H , Lin J , Luo P , Fu S , Cai H , Ye P , Xiao B , Mao W , Liu L , Yan Y , Liu M , Chen M , Zhang XJ , Wang X , Touyz RM , Xia J , Zhang BH , Huang X , Yuan Y , Rohit L , Liu PP , Li H . Association of inpatient use of angiotensin converting enzyme inhibitors and angiotensin II receptor blockers with mortality among patients with hypertension hospitalized with COVID‐19. Circ Res 2020;126:1671–1681. 32302265 10.1161/CIRCRESAHA.120.317134 PMC7265882

[21] Mancia G , Rea F , Ludergnani M , Apolone G , Corrao G. Renin‐angiotensin‐aldosterone system blockers and the risk of Covid‐19. N Engl J Med 2020;382:2431–2440. 32356627 10.1056/NEJMoa2006923 PMC7206933

[22] Julius S , Alderman MH , Beevers G , Dahlöf B , Devereux RB , Douglas JG , Edelman JM , Harris KE , Kjeldsen SE , Nesbitt S , Randall OS , Wright JT Jr . Cardiovascular risk reduction in hypertensive black patients with left ventricular hypertrophy: the LIFE study. J Am Coll Cardiol 2004;43:1047–1055. 15028365 10.1016/j.jacc.2003.11.029

[23] Taylor AL , Wright JT Jr . Should ethnicity serve as the basis for clinical trial design? Importance of race/ethnicity in clinical trials: lessons from the African‐American Heart Failure Trial (A‐HeFT), the African‐American Study of Kidney Disease and Hypertension (AASK), and the Antihypertensive and lipid‐Lowering Treatment to Prevent Heart Attack Trial (ALLHAT). Circulation 2005;112:3654–3660. 16330707 10.1161/CIRCULATIONAHA.105.540443

[24] Reynolds HR , Adhikari S , Pulgarin C , Troxel AB , Iturrate E , Johnson SB , Hausvater A , Newman JD , Berger JS , Bangalore S , Katz SD , Fishman GI , Kunichoff D , Chen Y , Ogedegbe G , Hochman JS . Renin‐angiotensin‐aldosterone system inhibitors and risk of Covid‐19. N Engl J Med 2020;382:2441–2448. 32356628 10.1056/NEJMoa2008975 PMC7206932

[25] Sama IE , Ravera A , Santema BT , van Goor H , Ter Maaten JM , Cleland JG , Rienstra M , Friedrich AW , Samani NJ , Ng LL , Dickstein K , Lang CC , Filippatos G , Anker SD , Ponikowski P , Metra M , van Veldhuisen DJ , Voors AA . Circulating plasma concentrations of angiotensin‐converting enzyme 2 in men and women with heart failure and effects of renin‐angiotensin‐aldosterone inhibitors. Eur Heart J 2020;41:1810–1817. 32388565 10.1093/eurheartj/ehaa373 PMC7239195

[26] de Simone G . Position statement of the ESC Council on Hypertension on ACE‐inhibitors and angiotensin receptor blockers. 13 March 2020. https://www.escardio.org/Councils/Council-on-Hypertension-(CHT)/News/position-statement-of-the-esc-council-on-hypertension-on-ace-inhibitors-and-ang (8 June 2020).

