# Supplementary material for: Angiotensin‐converting enzyme inhibitors and angiotensin II receptor blockers are not associated with severe COVID‐19 infection in a multi‐site UK acute hospital trust
Source: Eur J Heart Fail. 2020 Jul 7;22(6):967–74. doi: 10.1002/ejhf.1924 (PMC7301045; doi:10.1002/ejhf.1924)
Supplement: Supplementary file 1 — Table S1. Odds ratios and P‐values for all variables and primary endpoint. Figure S1. Performance of the CogStack and MedCAT NLP pipeline in detecting disease mentions within the electronic health record text. [file EJHF-22-967-s001.docx]

**Supplementary files**

| Model | Variable | OR (95% CI) | P-value |
| --- | --- | --- | --- |
| Baseline | On ACEi or ARB | 0.83 (0.64-1.07) | 0.16 |
| Model 1 | On ACEi or ARB | 0.70 (0.53-0.91) | <0.01 |
|  | Age (per 10 years) | 1.26 (1.17-1.36) | <0.01 |
|  | Male | 1.51 (1.18-1.93) | <0.01 |
| Model 2 | On ACEi or ARB | 0.64 (0.48-0.86) | <0.01 |
|  | Age (per 10 years) | 1.25 (1.16-1.35) | <0.01 |
|  | Male | 1.51 (1.18-1.94) | <0.01 |
|  | HTN | 1.22 (0.92-1.60) | 0.16 |
| Model 3 | On ACEi or ARB | 0.63 (0.47-0.84) | <0.01 |
|  | Age (per 10 years) | 1.24 (1.14-1.34) | <0.01 |
|  | Male | 1.50 (1.17-1.93) | <0.01 |
|  | HTN | 1.15 (0.86-1.55) | 0.34 |
|  | Diabetes | 1.07 (0.81-1.40) | 0.64 |
|  | HF or IHD | 0.95 (0.68-1.31) | 0.73 |
|  | CKD | 1.33 (0.95-1.86) | 0.09 |
| Hypertension unadjusted | HTN | 1.25 (0.98-1.59) | 0.069 |
| Hypertension adjusted | Age (per 10 years) | 1.24 (1.15-1.33) | <0.01 |
|  | Male | 1.50 (1.17-1.92) | <0.01 |
|  | HTN | 1.03 (0.80-1.32) | 0.83 |

**Supplementary Table 1.** **Odds ratios and p-values for all variables and primary endpoint.** Odds ratios and p-values calculated from logistic regressions. ACEi = Angiotensin converting enzyme inhibitor. OR = Odds ratio. ARB = Angiotensin Receptor Blocker. HTN = hypertension; HF = heart failure; IHD = ischaemic heart disease; CKD = chronic kidney disease.

**Supplementary Figure 1. Performance of the CogStack and MedCAT NLP pipeline in detecting disease mentions within the electronic health record text.** Precision (P), Recall (R) and F1 (harmonic mean of precision and recall). Only medical concept annotations with F1 > 80% and more than 10 annotated samples are shown. Disease names that start “Any: ” are aggregate concepts for multiple specific conditions that are used in our analysis.
